# Reporting and utilization of Patient-Reported Outcomes Measurement Information System® (PROMIS®) measures in orthopedic research and practice: a systematic review

**DOI:** 10.1186/s13018-020-02068-9

**Published:** 2020-11-23

**Authors:** Maggie E. Horn, Emily K. Reinke, Logan J. Couce, Bryce B. Reeve, Leila Ledbetter, Steven Z. George

**Affiliations:** 1 Duke Clinical Research Institute, Duke University, Durham, NC USA; 2 Department of Orthopaedic Surgery, Duke University, Box 10042, Durham, NC 27710 USA; 3 University of Utah Orthopaedic Center, University of Utah Health, Salt Lake City, UT USA; 4 Department of Population Health Sciences, Duke University School of Medicine, Durham, NC USA; 5 Department of Pediatrics, Duke University School of Medicine, Durham, NC USA; 6 Duke University Medical Center Library, Duke University, Durham, NC USA

**Keywords:** PROMIS, Patient-reported outcome measures, Orthopedics, Physical function, Pain

## Abstract

**Background:**

The Patient-Reported Outcomes Measurement Information System^Ⓡ^ (PROMIS^Ⓡ^) is a dynamic system of psychometrically sound patient-reported outcome (PRO) measures. There has been a recent increase in the use of PROMIS measures, yet little has been written about the reporting of these measures in the field of orthopedics. The purpose of this study was to conduct a systematic review to determine the uptake of PROMIS measures across orthopedics and to identify the type of PROMIS measures and domains that are most commonly used in orthopedic research and practice.

**Methods:**

We searched PubMed, Embase, and Scopus using keywords and database-specific subject headings to capture orthopedic studies reporting PROMIS measures through November 2018. Our inclusion criteria were use of PROMIS measures as an outcome or used to describe a population of patients in an orthopedic setting in patients ≥ 18 years of age. We excluded non-quantitative studies, reviews, and case reports.

**Results:**

Our final search yielded 88 studies published from 2013 through 2018, with 57% (50 studies) published in 2018 alone. By body region, 28% (25 studies) reported PROMIS measures in the upper extremity (shoulder, elbow, hand), 36% (32 studies) reported PROMIS measures in the lower extremity (hip, knee, ankle, foot), 19% (17 studies) reported PROMIS measures in the spine, 10% (9 studies) reported PROMIS measures in trauma patients, and 6% (5 studies) reported PROMIS measures in general orthopedic patients. The majority of studies reported between one and three PROMIS domains (82%, 73 studies). The PROMIS Computerized Adaptive Test (CAT) approach was most commonly used (81%, 72 studies). The most frequently reported PROMIS domains were physical function (81%, 71 studies) and pain interference (61%, 54 studies).

**Conclusion:**

Our review found an increase in the reporting of PROMIS measures over the recent years. Utilization of PROMIS measures in orthopedic populations is clinically appropriate and can facilitate communication of outcomes across different provider types and with reduced respondent burden.

**Registration:**

The protocol for this systematic review was designed in accordance with the PRISMA guidelines and is registered with the PROSPERO database (CRD42018088260).

**Supplementary Information:**

The online version contains supplementary material available at 10.1186/s13018-020-02068-9.

## Introduction

In order to determine if a patient has achieved a meaningful outcome, it is insufficient to evaluate treatment results solely on medical history, physical findings, laboratory tests, or imaging findings [1]. Patient-reported outcome (PRO) measures are a useful tool to quantify and communicate a patient’s health status to healthcare providers that directly incorporates the patient’s voice. Change in PROs can be one of the measures of “success” from a patient’s perspective after an orthopedic procedure [2]. PROs are increasingly being used as part of the clinical encounter to guide treatment decisions and determine the effectiveness of interventions [3], but PROs have presented challenges with implementation and measure selection.

In orthopedic practice and research, there is great variability in the number of PRO measures available. As a result, there is confusion among orthopedic providers about which PRO measure is most appropriate given a patient population and how to appropriately interpret a patient’s score to enhance treatment recommendations. Subsequently, in orthopedics, there has been a recent increase in the adoption of a universally accepted set of PRO measures: the Patient-Reported Outcomes Measurement Information System® (PROMIS®). PROMIS has been compared against conventional general health and disease-specific PRO measures and regularly has been found to improve coverage of the relevant health domain, increase reliability, and reduce respondent burden [4].

PROMIS measures were developed with support by the National Institutes of Health (NIH) as an effort to address the need for more valid, reliable, and generalizable measures of clinical outcomes that are important to patients [5]. PROMIS is a set of psychometrically sound measures to assess a patient’s physical, mental, and social health across multiple conditions or diseases, including orthopedic conditions. PROMIS measures overcome the limitations of traditional PRO measures used in orthopedic research and practice by scoring all PROMIS domains using a common metric of a T-score that is normalized to the U.S. general population. PROMIS provides access to both fixed-length measures (e.g., 6-item measure of fatigue) and computerized adaptive testing (CAT) that tailors the measure for each individual to allow for efficient assessment when response burden is of concern [6].

In recent years, a proliferation of studies have reported the association of PROMIS measures with traditional measures and have demonstrated the reliability and performance of PROMIS measures in orthopedic populations. While there have been a few systematic reviews about the use of PROMIS measures in certain disciplines within orthopedics [7–10], these reviews do not describe how the measures have been reported neither in the literature nor the general uptake of PROMIS measures within orthopedic research and practice. Thus, we sought to evaluate the adoption of PROMIS measures in orthopedics by describing how the measures are used and reported on, including the PROMIS domains evaluated, the type of PROMIS instrument used, and other traditional measures that were reported along with PROMIS measures.

## Methods

### Review design

The protocol for this systematic review was designed in accordance with the PRISMA guidelines [11] and is registered with the PROSPERO database (CRD42018088260) [12]. We collaborated with a research librarian (LL) to develop an appropriate search strategy and management of the literature review.

### Data sources and search strategy

We performed a literature search of PubMed, Embase, and Scopus from inception to November 4, 2018, using a combination of keywords and database-specific subject headings to capture studies done in an orthopedic setting and/or procedures that reported a PROMIS measure as an outcome (Additional file 1). We added search filters to exclude case studies or reports, editorials, letters to the editor, and studies not written in English.

### Inclusion and exclusion criteria

Inclusion criteria included the use of PROMIS measures in studies conducted in orthopedic settings for clinical care purposes or studies that used PROMIS measures to assess an outcome from an orthopedic intervention. Our exclusion criteria were study population < 18 years of age; non-orthopedic interventions, settings, or providers performing the intervention; and qualitative studies, commentaries, or systematic reviews. All included studies were peer-reviewed, reported at least one PROMIS measure, and used an experimental, quasi-experimental, or observational design. Two authors screened articles (MH and SZG) and a third author (ER) resolved any conflicts.

### Study selection and data extraction

After databases were searched, titles and abstracts of studies were uploaded into Covidence, a systematic review management software [13]. The article selection process was done in two phases. In the first phase, two authors (MH and SZG) performed independent reviews of titles and abstracts in Covidence using the predefined inclusion and exclusion criteria. Articles were moved to full-text review if one or both authors found the article potentially relevant. In the second phase, the same two authors independently reviewed full-text articles for eligibility. Any conflicts were resolved by the third author.

### Data analysis

Included studies were evaluated from November 2018 to June 2020. The primary purpose of this review was to describe the uptake of PROMIS measures in orthopedic research and practice through qualitative synthesis, and then rate the quality of included studies. Therefore, we did not perform a meta-analysis of data. For the qualitative synthesis, we described the studies by publication year, clinical population, study type, and sample size. We evaluated the reporting of PROMIS measures by recording the PRO domains reported in each study and the type of PROMIS measures used (i.e., domain-specific fixed short forms, multiple domain profile short forms, or CAT). Last, we described the frequency in which PROMIS measures were reported alongside traditional measures by the clinical population. Traditional measures are non-PROMIS established measures used in orthopedics.

### Quality assessment

We used the Newcastle-Ottawa Scale (NOS) to assess the quality of included studies (Additional file 2). Because this review included a heterogeneous group of studies with a wide variety of methodologies, there is likely no single risk of bias tool to perfectly evaluate study quality across such a diverse group. The NOS was developed to assess the quality of nonrandomized studies, and evaluates studies within three domains: the selection of study groups, the comparability between these groups, and the determination of the outcome of interest. We used a version of the NOS specifically adapted for cross-sectional studies [14] and for case control and cohort studies [15]. The NOS scoring of seven or more stars is generally considered high quality, though no ranges have been officially reported in the literature [16].

## Results

**Fig. 1:**
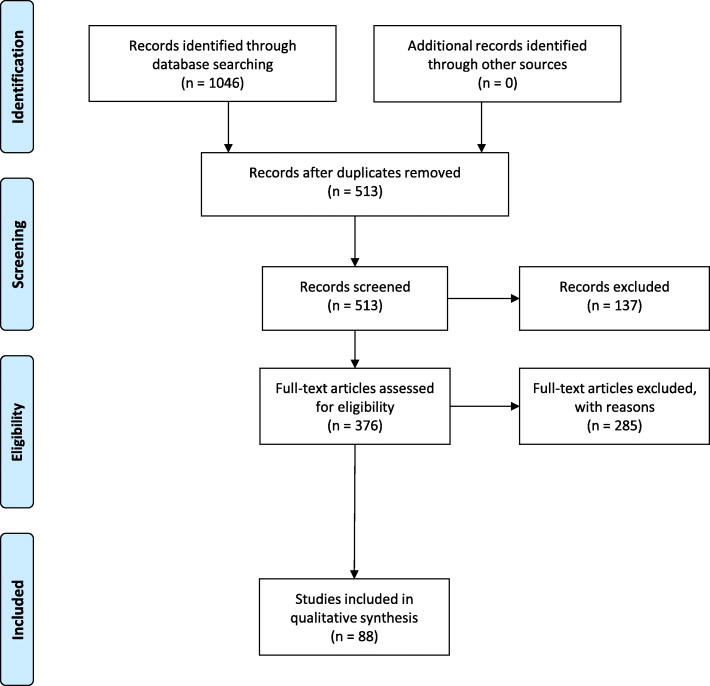
PRISMA literature flow diagram [11]

Our preliminary search yielded 1046 citations, and after duplicates were removed, 513 citations were reviewed by their titles and abstracts. Of those, 376 were moved forward to the full-text review stage, and 88 articles remained for inclusion in the systematic review [3, 17–103] (Fig. 1). After conflicts were resolved by the third author, we calculated an 81.6% agreement between the authors performing full-text review.

### Study characteristics

Table 1 shows the characteristics of included studies by year, clinical population, study type, and sample size.

**Table 1:** Study characteristics

Year	Author	Orthopedic population	Study design	***N***
2013	Hung et al. [17]	Lower extremity patients	Cross-sectional study	288
2014	Hung et al. [18]	Lower extremity patients	Cohort study (prospective observational study)	311
2014	Hung et al. [19]	Lower extremity patients	Cross-sectional study	126
2014	Hung et al. [3]	Trauma patients	Cross-sectional study	153
2014	Hunt et al. [20]	Lower extremity patients	Cohort study (prospective observational study)	140
2014	Papuga et al. [21]	Lower extremity patients	Cohort study (prospective observational study)	106
2014	Tyser et al. [22]	Upper extremity patients	Cross-sectional study	134
2015	Beckmann et al. [24]	Upper extremity patients	Cross-sectional study	187
2015	Mellema et al. [23]	Upper extremity patients	Cohort study (prospective observational study)	136
2015	Morgan et al. [25]	Trauma patients	Cross-sectional study	47
2015	Overbeek et al. [103]	Upper extremity patients	Cross-sectional study	93
2015	Stuart et al. [26]	Trauma patients	Cross-sectional study	55
2016	Beckmann et al. [29]	Upper extremity patients	Cross-sectional study	379
2016	Dasa et al. [33]	Lower extremity patients	Retrospective cohort	100
2016	Fuchs et al. [27]	Lower extremity patients	Retrospective cohort	93
2016	Hermanussen et al. [37]	Upper extremity patients	Cross-sectional study	111
2016	Ho et al. [28]	Lower extremity patients	Cohort study (prospective observational study)	61
2016	Nota et al. [30]	Upper extremity patients	Cross-sectional study	193
2016	Oak et al. [34]	Lower extremity patients	Cohort study (prospective observational study)	45
2016	Papuga et al. [35]	Spine patients	Cross-sectional study	319
2016	Parrish et al. [31]	Upper extremity patients	Cross-sectional study	112
2016	Peters et al. [32]	Upper extremity patients	Cross-sectional study	115
2016	van Leeuwen et al. [36]	Trauma patients	Cross-sectional study	124
2017	Anthony et al. [46]	Upper extremity patients	Cross-sectional study	70
2017	Anthony et al. [45]	Upper extremity patients	Cross-sectional study	82
2017	Beleckas et al. [41]	Upper extremity patients	Cohort study (prospective observational study)	5202
2017	Dowdle et al. [47]	Upper extremity patients	Cross-sectional study	53
2017	Hancock et al. [43]	Lower extremity patients	Cross-sectional study	107
2017	Henn et al. [53]	Upper extremity patients	Cohort study (prospective observational study)	300
2017	Kaat et al. [52]	Trauma patients	Cohort study (prospective observational study)	132
2017	Kazmers et al. [54]	Upper extremity patients	Cross-sectional study	1299
2017	Kleimeyer et al. [50]	Spine patients	Cohort study (prospective observational study)	88
2017	Koltsov et al. [38]	Lower extremity patients	Cohort study (prospective observational study)	191
2017	Nixon et al. [39]	Lower extremity patients	Cross-sectional study	85
2017	Oh et al. [40]	Upper extremity patients	Cross-sectional study	125
2017	Purvis et al. [49]	Spine patients	Cohort study (prospective observational study)	148
2017	Sheean et al. [42]	Lower extremity patients	Cross-sectional study	42
2017	St John et al. [55]	Upper extremity patients	Cross-sectional study	722
2018	Alvarez-Nebreda et al. [102]	Trauma patients	Cohort study (prospective observational study)	273
(101)	Anderson et al. [100]	Lower extremity patients	Cohort study (prospective observational study)	61
2018	Anderson et al. [101]	Lower extremity patients	Retrospective cohort	88
2018	Austin et al. [99]	Lower extremity patients	Retrospective cohort	2308
2018	Beleckas et al. [97]	General orthopedics	Cross-sectional study	14679
2018	Beleckas et al. [98]	General orthopedics	Retrospective cohort	3339
2018	Beleckas et al. [58]	Upper extremity patients	Cross-sectional study	3315
2018	Bernholt et al. [96]	Lower extremity patients	Retrospective cohort	75
2018	Bernstein et al. [95]	Lower extremity patients	Cohort study (prospective observational study)	500
2018	Bhatt et al. [94]	Spine patients	Cohort study (prospective observational study)	78
2018	Boody et al. [56]	Spine patients	Cohort study (prospective observational study)	59
2018	Cavallero et al. [93]	Trauma patients	Retrospective cohort	56
2018	Chen et al. [92]	Lower extremity patients	Retrospective cohort	233
2018	Crijns et al. [91]	Upper extremity patients	Retrospective cohort	4511
2018	Fisherauer et al. [59]	Upper extremity patients	Cross-sectional study	105
2018	Fram et al. [90]	Upper extremity patients	Retrospective cohort	11
2018	Gausden et al. [89]	Lower extremity patients	Cohort study (prospective Observational study)	132
2018	Gausden et al. [88]	Trauma patients	Cohort study (prospective observational study)	174
2018	Hancock et al. [87]	Lower extremity patients	Cross-sectional study	100
2018	Haskell et al. [61]	General orthopedics	Cross-sectional study	4524
2018	Haws et al. [86]	Spine patients	Retrospective cohort	74
2018	Hung et al. [85]	Lower extremity patients	Cohort study (prospective Observational study)	785
2018	Hung et al. [44]	Lower extremity patients	Cohort study (prospective observational study)	983
2018	Hung et al. [83]	Lower extremity patients	Cohort study (prospective observational study)	2226
2018	Hung et al. [84]	Lower extremity patients	Cohort study (prospective observational study)	3069
2018	Hung et al. [81]	Spine patients	Cohort study (prospective observational study)	763
2018	Hung et al. [82]	Spine patients	Cohort study (prospective observational study)	1945
2018	Hung et al. [60]	Upper extremity patients	Cross-sectional study	1759
2018	Kadri et al. 3 [80]	General orthopedics	Cross-sectional study	841
2018	Kagan et al. [79]	Lower extremity patients	Cohort study (prospective observational study)	91
2018	Karns et al. [78]	Lower extremity patients	Retrospective cohort	434
2018	Khechen et al. [77]	Spine patients	Retrospective cohort	41
2018	Kleimeyer et al. [76]	Spine patients	Retrospective cohort	75
2018	Kohring et al. [75]	Lower extremity patients	Retrospective cohort	271
2018	Kohring et al. [74]	Lower extremity patients	Retrospective cohort	540
2018	Kootstra et al. [73]	Upper extremity patients	Cross-sectional study	126
2018	Medina et al. [72]	General orthopedics	Cross-sectional study	937
2018	Meredith et al. [71]	Lower extremity patients	Cross-sectional study	383
2018	Merrill et al. [51]	Spine patients	Cohort study (prospective observational study)	111
2018	Nixon et al. [70]	Lower extremity patients	Retrospective cohort	159
2018	Owen et al. [48]	Spine patients	Cohort study (prospective observational study)	60
2018	Patel et al. [69]	Spine patients	Cohort study (prospective observational study)	98
2018	Patterson et al. [68]	Upper extremity patients	Cross-sectional study	164
2018	Patton et al. [67]	Lower extremity patients	Retrospective cohort	680
2018	Purvis et al. [66]	Spine patients	Cohort study (prospective observational study)	231
2018	Raad et al. [65]	Spine patients	Cohort study (prospective observational study)	76
2018	Rubery et al. [64]	Spine patients	Retrospective cohort	78
2018	Schwartz et al. [63]	Spine patients	Cohort study (prospective observational study)	167
2018	Stoop et al. [57]	Upper extremity patients	Cross-sectional study	122
2018	Vincent et al. [62]	Trauma patients	Cohort study (prospective Observational study)	101

#### Year

Studies included in this review were published from 2013 through 2018. The number of publications reporting PROMIS measures notably increased across time: 2013 (1%, 1 study), 2014 (7%, 6 studies), 2015 (6%, 5 studies), 2016 (13%, 11 studies), 2017 (17%, 15 studies). The majority of studies were published in 2018 (57%, 50 studies).

#### Clinical population

PROMIS measures were reported in orthopedic studies across multiple clinical populations. For reporting, we grouped the studies by body region rather than specific diagnosis. The majority of studies (36%, 32 studies) reported PROMIS measures in lower extremity disorders (hip, knee, ankle, foot), followed by upper extremity disorders (shoulder, elbow, hand) (28%, 25 studies), spine disorders (19%, 17 studies), orthopedic trauma (10%, 9 studies). Few studies (6%, 5 studies) reported PROMIS measures in general orthopedic patients.

#### Study type and sample size

The studies in this review varied in the study design used to assess outcomes. The largest percentage of studies were cohort studies (59%, 52 studies). Most of these were prospective observational designs (38%, 33 studies), and 22% (19 studies) were retrospective observational designs. Many studies (41%, 36 studies) used a cross-sectional study design to analyze the psychometric properties of PROMIS or to validate in a patient population. No randomized controlled trials were reported using PROMIS measures as an outcome measure. Sample sizes in the studies ranged from 11 patients to 14,679 patients, with 133 patients as the median number reported. Five studies included patients from registries including the American Orthopedic Foot and Ankle Society’s National Orthopedic Foot and Ankle Research Outcomes Network and the Maryland Orthopedic Registry.

### Reporting of PROMIS measures

The most frequently reported PROMIS domains in the studies included in this review were physical function (81%, 71 studies), pain interference (61%, 54 studies), depression (31%, 28 studies), physical function-upper extremity (18%, 16 studies), physical function-lower extremity (3%, 3 studies), and anxiety (15%, 13 studies) (Table 2). Most studies (75%, 66 studies) reported more than one PROMIS domain. Approximately a third of studies (32%, studies) reported two PROMIS domains, 25% (22 studies) reported three PROMIS domains, 9% (8 studies) reported four PROMIS domains, and the remainder (9%, 8 studies) reported between 5 and 9 PROMIS domains. Only a quarter (25%, 22 studies) reported one PROMIS domain. Of the type of PROMIS instrument used (i.e., CAT, short form, or profile), the vast majority of studies (81%, 71 studies) reported using the PROMIS CAT approach. A small percentage of studies reported only fixed-length instruments (15%, 13 studies) and (4%, 4 studies) reported a combination of CAT and fixed-length questionnaires.

**Table 2:** Reporting of PROMIS measures

Domain	Studies reporting domain	% CAT instrument format
Physical function	81% (71)	93%
Pain interference	61% (54)	85%
Pain behavior	4% (4)	100%
Emotional distress - depression	32% (28)	85%
Physical function - upper extremity	18% (16)	69%
Physical function - lower extremity	3% (3)	100%
Physical function - mobility	1% (1)	100%
Emotional support	1% (1)	100%
Psychological illness	2% (2)	100%
Instrumental support	1% (1)	100%
Sleep disturbance	4% (4)	50%
Emotional distress - anger	1% (1)	100%
Emotional distress - anxiety	13% (12)	83%
Fatigue	8% (7)	71%
Ability to participate in social roles and activities	1% (1)	100%
Satisfaction with participation in social roles	10% (9)	78%
Global health	7% (6)	0%
Pain intensity	4% (4)	0%
Emotional distress	1% (1)	0%

### PROMIS and traditional PROs

Fourteen studies in this review reported PROMIS as the sole outcome measure. Of those 14 studies, 9 were published in 2018 alone. Widely reported traditional measures were reported alongside PROMIS measures in all studies. Traditional measures included measuring the constructs of pain, disability, psychosocial comorbidity, and quality of life. Table 3 describes the reporting of traditional measures alongside PROMIS measures by body region.

**Table 3:** PROMIS domains and traditional PROs by body region

PROMIS domains/constructs	Traditional PRO measures
*General orthopedics*	
Physical function	International Knee Documentation Committee
Pain interference	American Shoulder and Elbow Surgeons Shoulder Score
Emotional distress—depression	Musculoskeletal Outcomes Data Evaluation and Management System
Emotional distress—anxiety	Tegner Activity Scale
Fatigue	Marx Activity Rating Scales
Satisfaction with participation in social roles	Brief Michigan Hand Questionnaire
Physical function—upper extremity	International Physical Activity Questionnaire
Pain intensity	Numeric Pain Scale—Global
Global health	Numeric Pain Scale—Local
Physical functional—lower extremity	
*Lower extremity*	
Physical function	Knee Injury and Osteoarthritis Outcome Score
Pain interference	Western Ontario and McMaster Universities Arthritis Index
Emotional distress—depression	Hip Disability and Osteoarthritis Outcome Score
Emotional distress—anxiety	Knee Injury and Osteoarthritis Outcome Score for Joint Replacement
Emotional distress—anger	Hip Disability and Osteoarthritis Outcome Score for Joint Replacement
Pain intensity	GAITRite Walk Testing
Fatigue	International Knee Documentation Committee
Satisfaction with participation in social roles	Oxford Knee Score
Sleep disturbance	Short Form 12
Pain behavior	Numeric Pain Scale—Global
Ability to participate in social roles and activities	Numeric Pain Scale—Local
Global health	Musculoskeletal Outcomes Data Evaluation and Management System
Physical function—mobility	Tegner Activity Scale
Physical function—upper extremity	Marx Activity Rating Scales
Physical functional—lower extremity	Short Form 36
	EuroQol EQ-5D
	Douleur Neuropathique 4 (DN4-I)
	Visual Analog Scale
	International Physical Activity Questionnaire
	Press Ganey Outpatient Medical Practice Survey
	Veterans RAND 12 (VR-12)
	Modified Harris Hip Score
	Posture Assessment Scale for Stroke
	Olerud-Molander Ankle Score
	Foot and Ankle Ability Measure
	Foot Function Index
	Foot and Ankle Outcome Score
	Short Form 36
	International Hip Outcome Tool (iHOT-33)
	Single Assessment Numeric Evaluation
	American Society of Anesthesiologists classification
*Spine*	
Physical function	Oswestry Disability Index
Pain interference	Neck Disability Index
Emotional distress—depression	Modified Japanese Orthopedic Association Scale
Emotional distress—anxiety	Short Form 12
Pain behavior	Global Rating of Change
Satisfaction with participation in social roles	Visual Analog Scale
Fatigue	EuroQol EQ-5D
Sleep disturbance	Scoliosis Research Society (SRS-22r)
Pain intensity	Generalized Anxiety Disorder (GAD-7)
Emotional distress	Patient Health Questionnaire for Depression Scale (PHQ-8)
	Short Form 36 (Rand-36 / SF-36)
	Zurich Claudication Questionnaire
	Brief Pain Inventory
	North America Spine Society Patient Satisfaction Index
	Coccygodynia Disability Index (CDI)
*Trauma*	
Physical function	Visual Analog Scale (VAS)
Pain intensity	Disabilities of the Arm, Shoulder, and Hand (DASH)
Physical function—upper extremity	Quick Disability of the Arm, Shoulder, and Hand (QuickDASH)
Satisfaction with participation in social roles	Constant Shoulder Score
Psychological illness	Short Musculoskeletal Functional Assessment (SMFA)
	Timed Up and Go
	Short Form 36 (Rand-36/SF-36)
	Injustice Experience Questionnaire
	Patient Health Questionnaire for Depression short form (PHQ-2)
	Pain Self-Efficacy Questionnaire short form (PSEQ-2)
	Pain Catastrophizing
	FRAIL Questionnaire
	UCLA Shoulder Score

### Quality of studies and risk of bias

A majority of studies assessed had a low risk of bias. All cohort and cross-sectional studies scored seven or above in their respective versions of the NOS quality assessment tool, and, with one exception, all case-control studies scored eight or above. Table 4 describes the risk of bias summary for individual studies included in this review, and Additional file 2 contains detailed results of the quality assessment.

**Table 4:** Risk of bias summary table

	# Studies	% Studies
Low (7 or above)	87	98.8%
Moderate to high (6 or below)	1	1.2%

## Discussion

In this review, we evaluated the uptake of PROMIS measures in orthopedic research and practice by describing how PROMIS measures were reported in published studies. The number of studies reporting the use of PROMIS measures increased exponentially from 2013 through 2017, with a spike in studies reporting PROMIS measures in 2018 alone (57% of total studies). This large increase in studies potentially indicates that PROMIS measures are being more widely adopted within orthopedic research and practice as an outcome measure. This increase may be due to the evolution of PROMIS measures from the short form, fixed instrument to the CAT instrument. Additionally, progress has been made with the availability and integration of PROMIS measures into Electronic Health Record (EHR) systems, allowing easier use of PROMIS CAT in the clinical setting [104, 105]. However, in relation to the increase in reporting of PROMIS measures in the literature, the vast majority of studies in our review reported the use of traditional measures alongside PROMIS measures [106]. This finding supports that, while PROMIS measures are gaining traction within orthopedics, researchers and clinicians may not be ready to abandon traditional measures in favor of PROMIS measures, despite evidence that the PROMIS domains of physical function and pain interference outperform traditional measures [107]. The reasons for this hesitancy may be related to familiarity with traditional measures, participation in registries that do not have PROMIS measures as part of the core set of measures, or a perceived lack of applicability in their patient populations. However, it may be noted that any new PRO measure should be considered experimental; thus, established measures are included both for validation purposes and to gain more understanding of how they relate to each other.

Our review also found that the use of PROMIS measures across clinical populations varied, with 37% of studies examining lower extremity conditions, followed by upper extremity (28%) and spine conditions (19%). This finding is consistent with the supporting literature where the use of PROMIS measures in lower, upper, and spine is increasing as a primary measure across clinical populations [1, 4, 108]. Last, most studies in our review reported the use of CAT-based assessments as the PROMIS assessment type. This finding is not surprising, as the primary benefits of the PROMIS CAT measures are the decrease in patient burden and the precision of the estimate. The majority of studies reported between one and three PROMIS domains. Unsurprisingly, the most commonly reported PROMIS domains were physical function and pain interference, which are validated and compared to many traditional measures. Of the psychological domains, depression was reported more frequently than anxiety. While the field of orthopedics is focused on improved functioning and reduced pain, we would encourage a more holistic view of the patient by incorporating more psychological constructs that may affect patient prognosis. This review provides evidence that the prevalence and support for use of PROMIS measures is growing in orthopedics and that PROMIS is being recognized as a PRO measure of choice for clinical trials [109].

### Limitations

Our systematic review has some limitations. First, we aimed to describe the prevalence and use of PROMIS measures within orthopedic practice and research rather than to compare outcomes or exposures in the studies. Our review had broad inclusion criteria, and thus there was high variability, with study designs often considered less rigorous. The majority of studies were retrospective and prospective cohort studies. No studies in our review were randomized clinical trials; however, this is likely because of the relative unavailability of PROMIS measures until recently. It will take some time before clinical trials that use PROMIS measures as endpoints are published.

Second, we reported on the PROMIS domains but did not perform meta-analyses to examine the effects of treatment or compare the performance of PROMIS measures with other reported measures. Last, many studies included in the review examined the reliability and validity of PROMIS measures in orthopedic populations, so the studies that reported PROMIS measures as the primary outcomes were less frequent, potentially leading to the impression that there is a higher prevalence of reporting PROMIS measures in the literature.

## Conclusions

PROMIS measures have been increasingly reported in orthopedic research and practice and present a new era of PRO measurement for clinical practice and scientific dissemination. Our findings are relevant for orthopedic researchers and clinicians who are using, or considering using, PROMIS measures. Our findings can provide guidance for stakeholders about the selection and administration of PRO measures, supporting value-based decisions both in clinics and prostheses procurement [110]. The domains of physical function and pain interference are the most commonly reported PROMIS domains, and these measure similar constructs to the traditional, body region-specific measures. Considerations about which PROMIS measures to administer in clinical populations should be made by determining what constructs are most important and whether PROMIS measures are sufficient alone or if traditional measures are needed to supplement the PROMIS measures. Given the evidence for the validity and reliability of PROMIS in orthopedics, we expect a decrease in the use of other established PRO measures in order to reduce respondent burden.

The implications for future research and practice in orthopedics support that PROMIS measures are versatile, reliable, and valid for orthopedic research and practice. Further, PROMIS measures provide distinct advantages over traditional measures, particularly, when the study population is heterogeneous. Multiple recent studies indicate that widespread variability exists in the particular PROs used in studies of the same diagnosis, thereby significantly limiting the translatability of many of these high-impact studies [6, 8, 111, 112]. Future research on the use of PROMIS measures in orthopedics should focus on the use of PROMIS measures as the primary outcome measure, particularly in studies that examine heterogeneous patient populations. Last, PROMIS measures hold immense potential for improving patient and provider communication, particularly across specialties.

## Supplementary information

Additional file 1: Search Strategies.

Additional file 2: Quality Assessment.

## Data Availability

A limited data set with fields reported in this paper is available upon request via email to the corresponding author, with no limitations on the reuse of the data.
