# Supplementary material for: Reporting and utilization of Patient-Reported Outcomes Measurement Information System® (PROMIS®) measures in orthopedic research and practice: a systematic review
Source: J Orthop Surg Res. 2020 Nov 23;15:553. doi: 10.1186/s13018-020-02068-9 (PMC7684926; doi:10.1186/s13018-020-02068-9)
Supplement: Supplementary file 1 — Search Strategies. [file 13018_2020_2068_MOESM1_ESM.docx]

**Appendix 1. Search Strategies**

Search: PROMIS

PubMed, Embase, Scopus

Date searched: November 4, 2018

**Database: Medline via Legacy PubMed**

| Set |  | Results |
| --- | --- | --- |
| 1 | ("Orthopedic Procedures"[Mesh] OR "Orthopedics"[Mesh] OR orthopedic[tiab] OR orthopaedic[tiab] OR orthopedics[tiab] OR orthopaedics[tiab] OR musculoskeletal[tiab] OR "neck"[MeSH Terms] OR neck[tiab] OR "spine"[MeSH Terms] OR spine[tiab] OR spinal[tiab] OR cervical[tiab] OR "lumbosacral region"[MeSH Terms] OR lumbosacral[tiab] OR lumbar[tiab] OR thoracic[tiab] OR "back"[MeSH Terms] OR back[tiab] OR "arthroplasty"[MeSH Terms] OR arthroplasty[tiab] OR "low back"[tiab] OR "lower back"[tiab] OR "shoulder"[MeSH Terms] OR shoulder[tiab] OR "elbow"[MeSH Terms] OR "elbow joint"[MeSH Terms] OR elbow[tiab] OR "hand"[MeSH Terms] OR hand[tiab] OR "hip"[MeSH Terms] OR hip[tiab] OR "knee"[MeSH Terms] OR "knee joint"[MeSH Terms] OR knee[tiab] OR "anterior cruciate ligament"[MeSH Terms] OR "anterior cruciate ligament"[tiab] OR trauma[tiab] OR "meniscus"[MeSH Terms] OR meniscus[tiab] OR "ankle"[MeSH Terms] OR "ankle joint"[MeSH Terms] OR ankle[tiab] OR "foot"[MeSH Terms] OR foot[tiab] OR "wrist"[MeSH Terms] OR "wrist joint"[MeSH Terms] OR wrist[tiab] OR "upper extremity"[MeSH Terms] OR "upper extremity"[tiab] OR "lower extremity"[MeSH Terms] OR "lower extremity"[tiab] OR "temporomandibular joint"[MeSH Terms] OR temporomandibular[tiab] OR "physiopathology"[Subheading] OR "Orthopedic Procedures"[Mesh] OR "Wounds and Injuries"[Mesh] OR "injuries"[Subheading] OR "Osteoarthritis"[Mesh] OR Osteoarthritis[tiab] OR "Arthritis"[Mesh] OR arthritis[tiab]) | 4152651 |
| 2 | (PROMIS[tiab] OR "patient reported outcome measurement information system"[tiab] OR "patient reported outcomes measurement information system"[tiab]) | 990 |
| 3 | #1 AND #2 | 364 |
| 4 | #3 NOT (Editorial[ptyp] OR Letter[ptyp] OR Comment[ptyp]) NOT (animals[mh] NOT humans[mh]) AND English[lang] | 359 |

**Database: Embase via Elsevier**

| Set |  | Results |
| --- | --- | --- |
| 1 | ('orthopedic surgery'/exp OR 'orthopedics'/exp OR orthopedic:ab,ti OR orthopaedic:ab,ti OR orthopedics:ab,ti OR orthopaedics:ab,ti OR musculoskeletal:ab,ti OR 'neck'/exp OR neck:ab,ti OR 'spine'/exp OR spine:ab,ti OR spinal:ab,ti OR cervical:ab,ti OR 'lumbosacral region'/exp OR lumbosacral:ab,ti OR lumbar:ab,ti OR thoracic:ab,ti OR 'back'/exp OR back:ab,ti OR 'arthroplasty'/exp OR arthroplasty:ab,ti OR ‘low back’:ab,ti OR ‘lower back’:ab,ti OR 'shoulder'/exp OR shoulder:ab,ti OR 'elbow'/exp OR elbow:ab,ti OR 'hand'/exp OR hand:ab,ti OR 'hip'/exp OR hip:ab,ti OR 'knee'/exp OR knee:ab,ti OR 'anterior cruciate ligament'/exp OR ‘anterior cruciate ligament’:ab,ti OR trauma:ab,ti OR 'knee meniscus'/exp OR meniscus:ab,ti OR 'ankle'/exp OR ankle:ab,ti OR 'foot'/exp OR foot:ab,ti OR 'wrist'/exp OR wrist:ab,ti OR 'upper limb'/exp OR ‘upper extremity’:ab,ti OR 'lower limb'/exp OR ‘lower extremity’:ab,ti OR 'temporomandibular joint'/exp OR temporomandibular:ab,ti OR 'pathophysiology'/exp OR 'orthopedic surgery'/exp OR 'injury'/exp OR 'osteoarthritis'/exp OR Osteoarthritis:ab,ti OR 'arthritis'/exp OR arthritis:ab,ti) | 5168555 |
| 2 | (PROMIS:ab,ti OR 'patient reported outcomes measurement information system'/exp OR ‘patient reported outcomes measurement information system’:ab,ti) | 1927 |
| 3 | #1 AND #2 | 641 |
| 4 | #3 NOT ('editorial'/exp OR 'letter'/exp OR 'note'/exp) AND [humans]/lim AND [english]/lim | 622 |

**Database: Scopus via Elsevier**

| Set |  | Results |
| --- | --- | --- |
| 1 | TITLE-ABS (orthopedic OR orthopaedic OR orthopedics OR orthopaedics OR musculoskeletal OR neck OR spine OR spinal OR cervical OR lumbosacral OR lumbar OR thoracic OR back OR arthroplasty OR "low back" OR "lower back" OR shoulder OR elbow OR hand OR hip OR knee OR "anterior cruciate ligament" OR trauma OR meniscus OR ankle OR foot OR wrist OR "upper extremity" OR "lower extremity" OR temporomandibular OR physiopathology OR injury OR injuries OR Osteoarthritis OR arthritis) | 4240383 |
| 2 | (PROMIS OR "patient reported outcomes measurement information system") | 1134 |
| 3 | #1 AND #2 | 284 |
| 4 | #3 AND ( LIMIT-TO ( DOCTYPE , "ar" ) OR LIMIT-TO ( DOCTYPE , "re" ) OR LIMIT-TO ( DOCTYPE , "ip" ) OR LIMIT-TO ( DOCTYPE , "ch" ) ) AND ( LIMIT-TO ( LANGUAGE , "English" ) ) | 269 |
