# Supplementary material for: Reporting and utilization of Patient-Reported Outcomes Measurement Information System® (PROMIS®) measures in orthopedic research and practice: a systematic review
Source: J Orthop Surg Res. 2020 Nov 23;15:553. doi: 10.1186/s13018-020-02068-9 (PMC7684926; doi:10.1186/s13018-020-02068-9)
Supplement: Supplementary file 2 — Quality Assessment. [file 13018_2020_2068_MOESM2_ESM.docx]

**Appendix 2. Quality Assessment**

| **PMID** | **Year** | **Author** | **Title** | **Study Type** | **Quality Assessment Tool** | **Quality Assessment Score** |
| --- | --- | --- | --- | --- | --- | --- |
| 24101733 | 2014 | Hung | Time for a paradigm shift with computerized adaptive testing of general physical function outcomes measurements | Cross-sectional study | NOS - Cross Sectional | **9** |
| 25161151 | 2014 | Hunt | The Orthopaedic Foot and Ankle Outcomes Research (OFAR) network: feasibility of a multicenter network for patient outcomes assessment in foot and ankle | Cohort study (prospective observational study) | NOS - Cohort | **7** |
| 24532421 | 2014 | Papuga | Validation of GAITRite and PROMIS as high-throughput physical function outcome measures following ACL reconstruction | Cohort study (prospective observational study) | NOS - Cohort | **8** |
| 24378399 | 2014 | Hung | Computerized Adaptive Testing Using the PROMIS Physical Function Item Bank Reduces Test Burden With Less Ceiling Effects Compared With the Short Musculoskeletal Function Assessment in Orthopaedic Trauma Patients | Cross-sectional study | NOS - Cross Sectional | **7** |
| 25099262 | 2015 | Overbeek | The PROMIS Physical Function Correlates With the QuickDASH in Patients With Upper Extremity Illness | Cross-sectional study | NOS - Cross Sectional | **9** |
| 26330786 | 2015 | Mellema | The effect of feedback regarding coping strategies and illness behavior on hand surgery patient satisfaction and communication: a randomized controlled trial | Cohort study (prospective observational study) | NOS - Cohort | **8** |
| 26001348 | 2015 | Morgan | PROMIS Physical Function Computer Adaptive Test Compared With Other Upper Extremity Outcome Measures in the Evaluation of Proximal Humerus Fractures in Patients Older Than 60 Years | Cross-sectional study | NOS - Cross Sectional | **8** |
| 26192378 | 2015 | Stuart | Reliability in Measuring Preinjury Physical Function in Orthopaedic Trauma | Cross-sectional study | NOS - Cross Sectional | **8** |
| 26231197 | 2016 | Fuchs | Effect of Arthroscopic Evaluation of Acute Ankle Fractures on PROMIS Intermediate-Term Functional Outcomes | Case-control study | NOS - Case-Control | **8** |
| 27530986 | 2016 | Ho | Preoperative PROMIS Scores Predict Postoperative Success in Foot and Ankle Patients | Cohort study (prospective observational study) | NOS - Cohort | **7** |
| 27172821 | 2016 | Nota | Is Social Support Associated With Upper Extremity Disability? | Cross-sectional study | NOS - Cross Sectional | **9** |
| 26718069 | 2016 | Parrish | Patient Satisfaction and its Relation to Perceived Visit Duration With a Hand Surgeon | Cross-sectional study | NOS - Cross Sectional | **9** |
| 26894216 | 2016 | Peters | Sleep Disturbance and Upper-Extremity Disability | Cross-sectional study | NOS - Cross Sectional | **9** |
| 26875052 | 2016 | Dasa | Percutaneous freezing of sensory nerves prior to total knee arthroplasty | Case-control study | NOS - Case-Control | **9** |
| 28210645 | 2016 | Oak | Responsiveness Comparison of the EQ-5D, PROMIS Global Health, and VR-12 Questionnaires in Knee Arthroscopy | Cohort study (prospective observational study) | NOS - Cohort | **8** |
| 26909832 | 2016 | Papuga | Correlation of PROMIS Physical Function and Pain CAT Instruments With Oswestry Disability Index and Neck Disability Index in Spine Patients | Cross-sectional study | NOS - Cross Sectional | **8** |
| 26994517 | 2016 | van Leeuwen | Does perceived injustice correlate with pain intensity and disability in orthopaedic trauma patients? | Cross-sectional study | NOS - Cross Sectional | **9** |
| 27847850 | 2016 | Hermanussen | Predictors of Upper-Extremity Physical Function in Older Adults | Cross-sectional study | NOS - Cross Sectional | **9** |
| 28836098 | 2017 | Nixon | PROMIS Pain Interference and Physical Function Scores Correlate With the Foot and Ankle Ability Measure (FAAM) in Patients With Hallux Valgus | Cross-sectional study | NOS - Cross Sectional | **8** |
| 28344534 | 2017 | Oh | The Influence of Psychological Factors on the Michigan Hand Questionnaire | Cross-sectional study | NOS - Cross Sectional | **9** |
| 28609640 | 2017 | Sheean | Assessment of Disability Related to Femoroacetabular Impingement Syndrome by Use of the Patient-Reported Outcome Measure Information System (PROMIS) and Objective Measures of Physical Performance | Cross-sectional study | NOS - Cross Sectional | **9** |
| 28590381 | 2017 | Hancock | Performance of PROMIS for Healthy Patients Undergoing Meniscal Surgery | Cross-sectional study | NOS - Cross Sectional | **9** |
| 28655475 | 2017 | Anthony | Preoperative Performance of the Patient-Reported Outcomes Measurement Information System in Patients With Rotator Cuff Pathology | Cross-sectional study | NOS - Cross Sectional | **9** |
| 28944248 | 2017 | Dowdle | Use of PROMIS for Patients Undergoing Primary Total Shoulder Arthroplasty | Cross-sectional study | NOS - Cross Sectional | **9** |
| 28787313 | 2018 | Owen | PROMIS Physical Function Correlation with NDI and mJOA in the Surgical Cervical Myelopathy Patient Population | Cohort study (prospective observational study) | NOS - Cohort | **8** |
| 28800569 | 2017 | Kleimeyer | Surgery for Refractory Coccygodynia | Case-control study | NOS - Case-Control | **9** |
| 28704333 | 2018 | Merrill | The Impact of Depression on Patient Reported Outcome Measures After Lumbar Spine Decompression | Case-control study | NOS - Case-Control | **9** |
| 29062209 | 2017 | Henn | The Maryland Orthopaedic Registry (MOR): Design and baseline characteristics of a prospective registry | Cohort study (prospective observational study) | NOS - Cohort | **7** |
| 28890332 | 2017 | Kazmers | Association of Physical Function, Anxiety, and Pain Interference in Nonshoulder Upper Extremity Patients Using the PROMIS Platform | Cross-sectional study | NOS - Cross Sectional | **8** |
| 28709796 | 2017 | St John | Efficacy of PROMIS Pain Interference and Likert Pain Scores to Assess Physical Function | Cross-sectional study | NOS - Cross Sectional | **9** |
| 28718322 | 2018 | Stoop | The PROMIS Global Health Questionnaire Correlates With the QuickDASH in Patients With Upper Extremity Illness | Cross-sectional study | NOS - Cross Sectional | **9** |
| 29395589 | 2018 | Beleckas | Relative Prevalence of Anxiety and Depression in Patients With Upper Extremity Conditions | Cross-sectional study | NOS - Cross Sectional | **9** |
| 29175295 | 2018 | Fisherauer | Pain anxiety differentially mediates the association of pain intensity with function depending on level of intolerance of uncertainty | Cross-sectional study | NOS - Cross Sectional | **9** |
| 29366343 | 2018 | Haskell | Implementation of Patient-Reported Outcomes Measurement Information System Data Collection in a Private Orthopedic Surgery Practice | Cross-sectional study | NOS - Cross Sectional | **8** |
| 30130305 | 2018 | Vincent | Patient-Reported Outcomes Measurement Information System Outcome Measures and Mental Health in Orthopaedic Trauma Patients During Early Recovery | Cohort study (prospective observational study) | NOS - Cohort | **8** |
| 30248391 | 2018 | Schwartz | Reconsidering the minimally important difference: evidence of instability over time and across groups | Cohort study (prospective observational study) | NOS - Cohort | **7** |
| 30074972 | 2018 | Rubery | Preoperative PROMIS Scores Assist in Predicting Early Postoperative Success in Lumbar Discectomy | Case-control study | NOS - Case-Control | **9** |
| 29807793 | 2018 | Patton | Clinical Outcomes of Patients With Lateral Femoral Cutaneous Nerve Injury After Direct Anterior Total Hip Arthroplasty | Case Series | NOS - Case-Control | **9** |
| 29776469 | 2018 | Patterson | Correlation of Patient-Reported Outcomes Measurement Information System (PROMIS) scores with legacy patient-reported outcome scores in patients undergoing rotator cuff repair | Cross-sectional study | NOS - Cross Sectional | **8** |
| 29648889 | 2018 | Nixon | Patient-Reported Allergies Do Not Predict Poorer PROMIS Function, Pain, and Depression Scores Following Foot and Ankle Surgery | Case-control study | NOS - Case-Control | **9** |
| 29991075 | 2018 | Meredith | Preoperative Opioid Use in Knee Surgery Patients | Cross-sectional study | NOS - Cross Sectional | **9** |
| 30135987 | 2018 | Medina | Orthopaedic surgery patients who use recreational marijuana have less pre-operative pain | Cross-sectional study | NOS - Cross Sectional | **9** |
| 29481341 | 2018 | Kootstra | Is Physician Empathy Associated With Differences in Pain and Functional Limitations After a Hand Surgeon Visit? | Cross-sectional study | NOS - Cross Sectional | **8** |
| 29691167 | 2018 | Kohring | Press Ganey Outpatient Medical Practice Survey Scores Do Not Correlate With Patient-Reported Outcomes After Primary Joint Arthroplasty | Case-control study | NOS - Case-Control | **9** |
| 29506925 | 2018 | Kohring | Treated Versus Untreated Depression in Total Joint Arthroplasty Impacts Outcomes | Case-control study | NOS - Case-Control | **9** |
| 29529003 | 2018 | Kleimeyer | Selective Anterior Lumbar Interbody Fusion for Low Back Pain Associated With Degenerative Disc Disease Versus Nonsurgical Management | Case-control study | NOS - Case-Control | **9** |
| 30094271 | 2018 | Karns | Patient- and Procedure-Specific Variables Driving Total Direct Costs of Outpatient Anterior Cruciate Ligament Reconstruction | Case-control study | NOS - Case-Control | **9** |
| 29656980 | 2018 | Kagan | The Recovery Curve for the Patient-Reported Outcomes Measurement Information System Patient-Reported Physical Function and Pain Interference Computerized Adaptive Tests After Primary Total Knee Arthroplasty | Cohort study (prospective observational study) | NOS - Cohort | **8** |
| 30140710 | 2018 | Kadri | How Long Does It Take for Patients to Complete PROMIS Scores? An Assessment of PROMIS CAT Questionnaires Administered at an Ambulatory Sports Medicine Clinic | Cross-sectional study | NOS - Cross Sectional | **8** |
| 29974172 | 2018 | Hancock | PROMIS: a valid and efficient outcomes instrument for patients with ACL tears | Cross-sectional study | NOS - Cross Sectional | **8** |
| 29742949 | 2018 | Fram | Surgical transposition for chronic instability of the extensor carpi ulnaris tendon | Case-control study | NOS - Case-Control | **4** |
| 29908926 | 2018 | Crijns | Factors Associated With a Discretionary Upper-Extremity Surgery | Case-control study | NOS - Case-Control | **9** |
| 29761114 | 2018 | Chen | Preoperative PROMIS Scores Predict Postoperative Outcomes After Primary ACL Reconstruction | Case-control study | NOS - Case-Control | **9** |
| 29738401 | 2018 | Cavallero | Locking Plate Fixation in a Series of Bicondylar Tibial Plateau Fractures Raises Treatment Costs Without Clinical Benefit | Case-control study | NOS - Case-Control | **9** |
| 30282475 | 2018 | Bernstein | PROMIS Pain Interference Is Superior vs Numeric Pain Rating Scale for Pain Assessment in Foot and Ankle Patients | Cohort study (prospective observational study) | NOS - Cohort | **8** |
| 29740783 | 2018 | Beleckas | Anxiety in the orthopedic patient: using PROMIS to assess mental health | Cross-sectional study | NOS - Cross Sectional | **9** |
| 30216242 | 2018 | Beleckas | Using Patient-reported Outcomes Measurement Information System Measures to Understand the Relationship Between Improvement in Physical Function and Depressive Symptoms | Case-control study | NOS - Case-Control | **9** |
| 30100133 | 2018 | Austin | Patient Outcomes After Total Knee Arthroplasty in Patients Older Than 80 Years | Case-control study | NOS - Case-Control | **9** |
| 29620940 | 2018 | Anderson | Validation and Generalizability of Preoperative PROMIS Scores to Predict Postoperative Success in Foot and Ankle Patients | Cohort study (prospective observational study) | NOS - Cohort | **8** |
| 29791196 | 2018 | Anderson | Determining Success or Failure After Foot and Ankle Surgery Using Patient Acceptable Symptom State (PASS) and Patient Reported Outcome Information System (PROMIS) | Case-control study | NOS - Case-Control | **9** |
| 30256341 | 2018 | Alvarez-Nebreda | Reliability of Proxy-reported Patient-reported Outcomes Measurement Information System Physical Function and Pain Interference Responses for Elderly Patients With Musculoskeletal Injury | Cohort study (prospective observational study) | NOS - Cohort | **8** |
